# Derricin and Derricidin Inhibit Wnt/β-Catenin Signaling and Suppress Colon Cancer Cell Growth *In Vitro*

**DOI:** 10.1371/journal.pone.0120919

**Published:** 2015-03-16

**Authors:** Barbara F. Fonseca, Danilo Predes, Debora M. Cerqueira, Alice H. Reis, Nathalia G. Amado, Marina C. L. Cayres, Ricardo M. Kuster, Felipe L. Oliveira, Fabio A. Mendes, Jose G. Abreu

**Affiliations:** 1 Universidade Federal do Rio de Janeiro, Instituto de Ciências Biomédicas, Rio de Janeiro, Brazil; 2 Universidade Federal do Rio de Janeiro, Instituto de Pesquisas em Produtos Naturais, Rio de Janeiro, Brazil; University of Kentucky, UNITED STATES

## Abstract

Overactivation of the Wnt/β-catenin pathway in adult tissues has been implicated in many diseases, such as colorectal cancer. Finding chemical substances that can prevent this phenomenon is an emerging problem. Recently, several natural compounds have been described as Wnt/β-catenin inhibitors and might be promising agents for the control of carcinogenesis. Here, we describe two natural substances, derricin and derricidin, belonging to the chalcone subclass, that show potent transcriptional inhibition of the Wnt/β-catenin pathway. Both chalcones are able to affect the cell distribution of β-catenin, and inhibit Wnt-specific reporter activity in HCT116 cells and in *Xenopus* embryos. Derricin and derricidin also strongly inhibited canonical Wnt activity *in vitro*, and rescued the Wnt-induced double axis phenotype in *Xenopus* embryos. As a consequence of Wnt/β-catenin inhibition, derricin and derricidin treatments reduce cell viability and lead to cell cycle arrest in colorectal cancer cell lines. Taken together, our results strongly support these chalcones as novel negative modulators of the Wnt/β-catenin pathway and colon cancer cell growth *in vitro*.

## Introduction

Wnt/β-catenin signaling is the main cause of many different neoplasias, and has a close association with colorectal cancer (CRC). Approximately 80% of all CRCs have mutations in Wnt components, which results in overactivation of the pathway in colon cells. The most common mutations result in APC loss of function which are negative regulators of this signaling pathway; and gain of function mutations in β-catenin, the main effector protein of this signaling [1,2,3,4]. These mutations cause uncontrolled overexpression of several oncogenes and cell cycle genes, particularly in cells derived from the intestinal crypts [5].

CRC is a malignant disease with high prevalence, and the third most commonly diagnosed cancer in the world [6]. It is difficult to treat; surgery and adjuvant drugs are commonly used, but cure only a small percentage of tumors [7]. CRC is strongly associated with genetic disorders, which is a result of mutations in important cell cycle and apoptosis regulatory genes, such as KRAS, TP53 and BRAF [8]. In addition, CRC can also be result of human nutritional patterns. It has been shown that high consumption of plant-origin food is related to a reduction of CRC incidence, because of the large amounts of natural antitumor compounds such as flavonoids and other polyphenols [7,9].

Flavonoids are polyphenolic compounds found in many plants and have a wide range of biological effects. Because flavonoids are a large part of the human diet, they have been intensively studied in regard to their beneficial effects in many human diseases, such as cancer. They have anti-proliferative, anti-invasive and pro-apoptotic effects [10,11,12]. Many flavonoids described as Wnt inhibitors have interesting effects on CRC control and also contribute to the prevention of this disease [13,14,15,16,17]. The flavonoids EGCG and quercetin have been reported as promising anti-cancer drugs [18,19]. Among the effects of these flavonoids is the regulation of signaling pathways that are closely associated with cancer, such as Wnt/β-catenin signaling, although none of them has succeeded as an effective drug for cancer treatment.

Here, we investigated the effect of two little-known flavonoids, derricin and derricidin, which were extracted from *Lonchocarpus sericeus* [20]. Derricin and derricidin are able to reduce CRC growth *in vitro*, by controlling the cell cycle progression. Moreover, derricin and derricidin strongly inhibited Wnt8-induced double axis in *Xenopus* embryos, which strongly indicates that these flavonoids are modulators of the Wnt/β-catenin pathway.

## Materials and Methods

### Cell lines, chemicals and reagents

All cell culture reagents were purchased from Gibco-Invitrogen (Carlsbad, CA, USA). Dimethyl sulfoxide (DMSO) and anti-β-catenin were purchased from Sigma (St. Louis, MO, USA). Secondary antibodies were purchased from Life Technologies (CA, USA). Cell lines used were HEK293t, L-cell, L-Wnt3a, HCT116, DLD-1 and IEC-18 (ATCC) and RKO-pBAR/*Renilla* [21]. The chalcones derricin and derricidin used in this study were extracted and purified by Nascimento and Mors (1972) [20].

### Wnt-Luciferase reporter Assays

RKO-pBAR/*Renilla* cells were cultured on 96-well plates, with 1.0 x 10^4^ cells/well in DMEM-High Glucose with 10% fetal bovine serum (Gibco). After confluence, cells were treated with derricin (10, 20 or 50 μM) or derricidin (10, 20 or 50 μM) in the presence of Wnt3a conditioned medium [22], for an additional 24 h. L-cell conditioned medium was used as negative control. DMSO was also added as the vehicle control. After 24 h of treatment, Firefly and *Renilla* luciferase activities were detected according to the manufacturer’s protocol (Dual Luciferase Reporter Assay System, Promega).

HEK293t and HCT116 were cultured on 96-well plates with 1.0 x 10^4^ cells/well in DMEM-F12 with 10% fetal bovine serum (Gibco). After 70% confluence was reached, each well was transfected with 50 ng TOP-Flash or FOP-Flash plasmids, 5 ng TK-*Renilla*-luciferase, with or without β-catenin [23]. The transfection reagent used was Lipofectamine (Invitrogen). 15h after transfection, cells were treated with chalcones in the presence of Wnt3a conditioned medium, for 24 h, using 10, 20 or 30 μM of derricin or 10, 20 or 30 μM of derricidin. On the next day, Firefly and *Renilla* luciferase activities were detected according to the manufacturer’s protocol (Dual Luciferase Reporter Assay System, Promega).

#### Embryo Manipulations

Frog experiments were carried out according to the guidelines granted by the Animal Care and Use Ethic Committee (Comissão de Ética no Uso de Animais—CEUA) of the Federal University of Rio de Janeiro and were approved by this committee under the permission number 152/13. Adult frogs (Nasco Inc., WI, USA) were stimulated with human chorionic gonadotropin (Sigma, St. Louis, MO, USA). *Xenopus* embryos were obtained by *in vitro* fertilization and staged according to Nieuwkoop and Farber [24]. All experiments were performed at 22°C. For synthetic xWnt8 mRNA, the plasmid was linearized with NotI and transcribed with SP6 RNA polymerase using the mMessage mMachine kit (Applied Biosystems). Four-cell-stage embryos were injected into the ventral marginal zone in order to induce secondary axis formation. In addition, four-cell-stage embryos were co-injected with 10 pg/embryo of xWnt8 mRNA plus 0.4 pmol/embryo of each chalcone or 250 pg of Wnt/β-catenin luciferase reporter plasmid (S01234-Luc) and 50 pg TK-*Renilla* to perform the embryo luciferase assays. After injection, embryos were maintained in 0.1x Barth (8.89 mM NaCl; 0.1 mM KCl; 0.24 mM NaHCO_3_; 0.08 mM MgSO_4_.7H_2_O; 1 mM Hepes; 0.03 mM Ca(NO_3_)_2_.4H_2_O; 0.04 mM CaCl_2_.2H_2_O; pH 7.7), until stage 27, when the phenotypes were analyzed or until gastrula stage (st 10) when the luciferase activity was detected according to the manufacturer’s protocol (Dual Luciferase Reporter Assay System, Promega).

#### MTT assay

3-(4,5-Dimethylthiazol-2-yl)-2,5-diphenyl tetrazolium bromide (MTT) was used to assay mitochondrial activity in viable cells. Cells were plated at a concentration of 1.0 x 10^4^ cells/well in 96-well tissue culture plates in DMEM F-12 medium containing 10% fetal bovine serum and cultured for 24 h before treatment with chalcones (10, 20, 30, 50, or 100 μM) for 0, 24, 48, or 72 h. MTT was added to each well at a final concentration of 150 mg/ml for 4 h before cell harvesting. The formazan reaction product was dissolved with DMSO and quantified spectrophotometrically at 570 nm (Modulus II microplate multimode reader).

#### Immunostaining

HCT116 cells were fixed in 4% paraformaldehyde, washed with PBS, and permeabilized with 0.1% Triton X-100. Samples were then blocked for 1 h with 5% bovine serum albumin. A rabbit anti-β-catenin (1:200) primary antibody was incubated overnight. Specific secondary antibodies conjugated with Cy3 fluorochrome (1:5000) were incubated for 2 h at room temperature. After PBS washes, DAPI staining (4,6-diamidino-2-phenylindole) (Cell Signaling) was performed for 5 min, and then slides were mounted with FluorSave (Calbiochem) and observed in a Nikon TE 2000-S inverted microscope (Melville, NY, USA). Images were captured using a CoolSNAP-Pro (Media Cybernetics, Bethesda, MD, USA) digital camera, with a zoom of 100x and 600x.

### Cell proliferation assay

For cell proliferation assay, 5,0 x10^4^ cells were plated on the previous day and treated with 30 μM or 50 μM derricin or derricidin for 24 h. Click-iT EdU (Life Sciences) assay was performed according to manufacturer’s protocol. DMSO was used as a vehicle to solubilize the flavonoids and was added to control cultures conditions at 0,5%.

#### Cell cycle analysis

Cell cycle analysis was performed using flow cytometry. Cells were rinsed briefly with calcium and PBS and detached with trypsin at room temperature. After centrifugation, 1×10^6^ cells were suspended in 0.5 mL ice-cold VindeLov solution [25] containing 0.1% Triton X-100, 0.1% citrate buffer, 0.1 mg/ml RNase and 50 μg/mL propidium iodide (Sigma Chemical Co., St. Louis, MO, USA). After 15 min, the cells were analyzed for DNA content using a FACSCalibur flow cytometer (Becton Dickinson, Mountain View, CA, USA). Living cells were analyzed according DNA-content. Cells showing fragmented DNA and possible doublets were excluded from the analysis. The cells with diploid DNA content (2n, G0–G1 phase), synthesizing DNA (>2n but <4n, S phase), and duplicated DNA (4n, G2/M phase) were acquired and analyzed using CellQuest and WinMDI 2.9 software, respectively.

#### Statistical analysis

Each experiment was performed at least three times. The luciferase, cell cycle and MTT assays were performed in triplicate. Cell staining quantification was performed by counting the number of DAPI and the number of non-nuclear and nuclear β-catenin stained cells in randomly chosen microscope fields, and then the percentage of non-nuclear and nuclear cells of the total cells was calculated. Statistical analysis was performed using the unpaired Student’s t test. In the MTT assays, we used two-way ANOVA following a Bonferroni post-test (GraphPad Prism version 5.00, GraphPad Software Inc., La Jolla, CA, USA). In the *Xenopus* Wnt/β-catenin reporter assay we used one-way ANOVA following a Kruskal-Wallis (GraphPad Prism version 6.00, GraphPad Software Inc., La Jolla, CA, USA). Statistical significance set at *P<0.05, **P<0.01, and ***P<0.001.

## Results

### Derricin and derricidin inhibit the proliferation of CRCs cell lines

**Fig 1:**
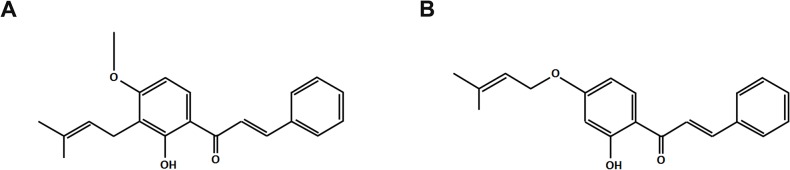
Chemical structures of the chalcones derricin (A) and derricidin (B).

**Fig 2:**
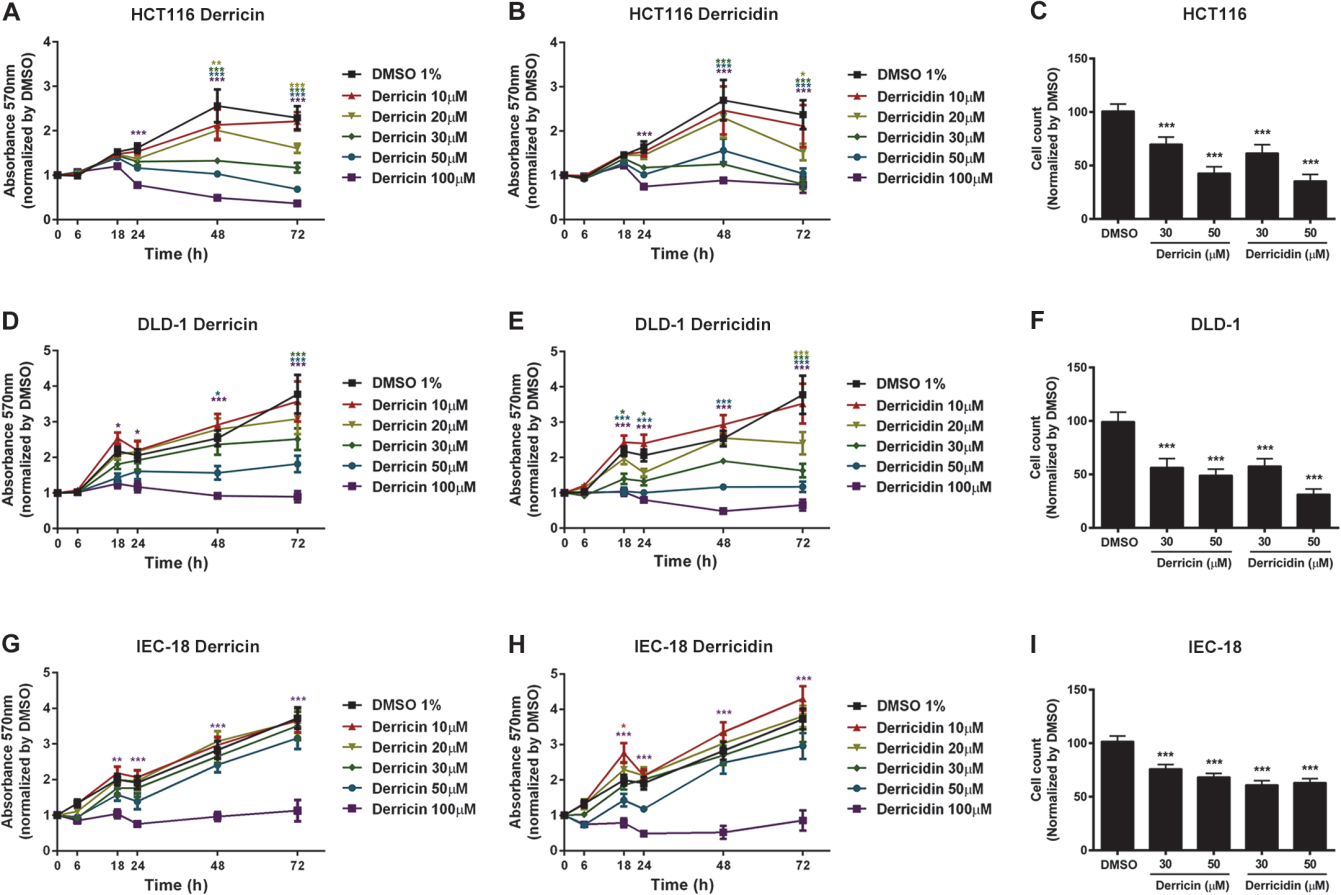
Derricin and derricidin reduce cell number and cell viability of colon cells. (A, B, D, E, G, H) Graphs show MTT absorbance levels in HCT116, DLD-1 and IEC-18 cell lines, after treatment up to 72 h with 10–100 μM of derricin or derricidin. (A) In HCT116 cells, it is observed significant reduction in cell viability after 100 μM of derricin for 24 h and 20 μM of derricin for 48 h (B) After derricidin treatment, reduction of cell viability occurred at 100 μM of derricidin after 24 h and at 30 μM after 72 h. (D) In DLD-1 cells, it is observed a milder effect at 100 μM after 18h of treatment. Otherwise, an intense reduction of MTT absorbance is detected after 48h treatment with 100 μM of derricin. (E) Derricidin treatment significantly reduces DLD-1 cell viability after 18h treatment with 50 μM of flavonoid and at 20 μM after 72h. (G) In IEC-18 cells, reduction of viability is observed with 100 μM of derricin, starting at 18h of treatment. (H) A similar profile is observed after derricidin treatment. (C,F,I) DAPI-stained nuclei were counted after 24h treatment with derricin and derricidin. (C) 30 and 50μM of derricin reduced approximately 30% and 58% the number of HCT116 nuclei, respectively; while derricidin reduced approximately 39% and 65%, respectively. (F) DLD-1 nuclei were decreased about 44% and 50% after derricin treatment at 30 and 50 μM concentrations, respectively. Derricidin treatment diminished about 50% and 70%, respectively. (I) IEC-18 nuclei were also decreased, with values of 25% and 32% of reduction with 30 and 50μM of derricin, respectively, and 40% and 38% with 30 and 50μM of derricidin, respectively. * p<0.05, **p<0.01, ***p<0.001.

Many natural compounds, including flavonoids, are able to affect cell proliferation and apoptosis and thus are important candidates for cancer control. We performed a low throughput Wnt specific reporter assay to screen natural compounds extracted from different Brazilian plants (data not shown). Among the inhibitors, we found a detectable Wnt/β-catenin inhibition activity displayed by the chalcones derricin and derricidin (Fig. 1). Here, we used two CRC cell lines, HCT116 and DLD-1, and investigated if derricin and derricidin could affect colon cells growth. We also investigated the chalcones effect in IEC-18, a non-transformed cell line derived from rat intestine epithelium. First, we treated these cells at different concentrations (10–100 μM) of these chalcones for different treatment periods (0–72 h), and analyzed cell viability, through MTT assay. In HCT116, we observed a significant decrease in cell viability at 100 μM of derricin in the first 24 hours of treatment. After 48 h of treatment, 20 μM of derricin was able to reduce MTT absorbance (Fig. 2A). Derricidin had similar effects on HCT116 and also reduced cell viability in a concentration- and time-dependent manner (Fig. 2B). Moreover, treatment with derricin and derricidin decreased the number of HCT116 cells, achieving approximately 58% and 65% of reduction of DAPI-stained nuclei with 50 μM of derricin and derricidin, respectively (Fig. 2C). In DLD-1, derricin could reduce cell viability at 100 μM concentration with a milder effect at 18 h and an intense reduction after 48 h of treatment (Fig. 2D). Derricidin, on the other hand, had a stronger effect in DLD-1, as 50 μM of this chalcone was sufficient to strongly decrease cell viability after 18 h of treatment (Fig. 2E). These chalcones were also able to decrease the number of DLD-1 cells in culture, achieving approximately 50% and 70% of reduction with 50 μM of derricin and derricidin, respectively (Fig. 2F). When we analyzed the effect of derricin and derricidin in cell viability of IEC-18 cells, we could observe that both flavonoids decrease MTT absorbance, starting at 18 h of treatment at 100μM. Interestingly, no effects in cell viability were detected at lower concentrations (Fig. 2G,H). Moreover, 50 μM of derricin and derricidin treatment resulted in a maximal reduction of 32% and 38% of IEC-18 cell number (Fig. 2I).

**Fig 3:**
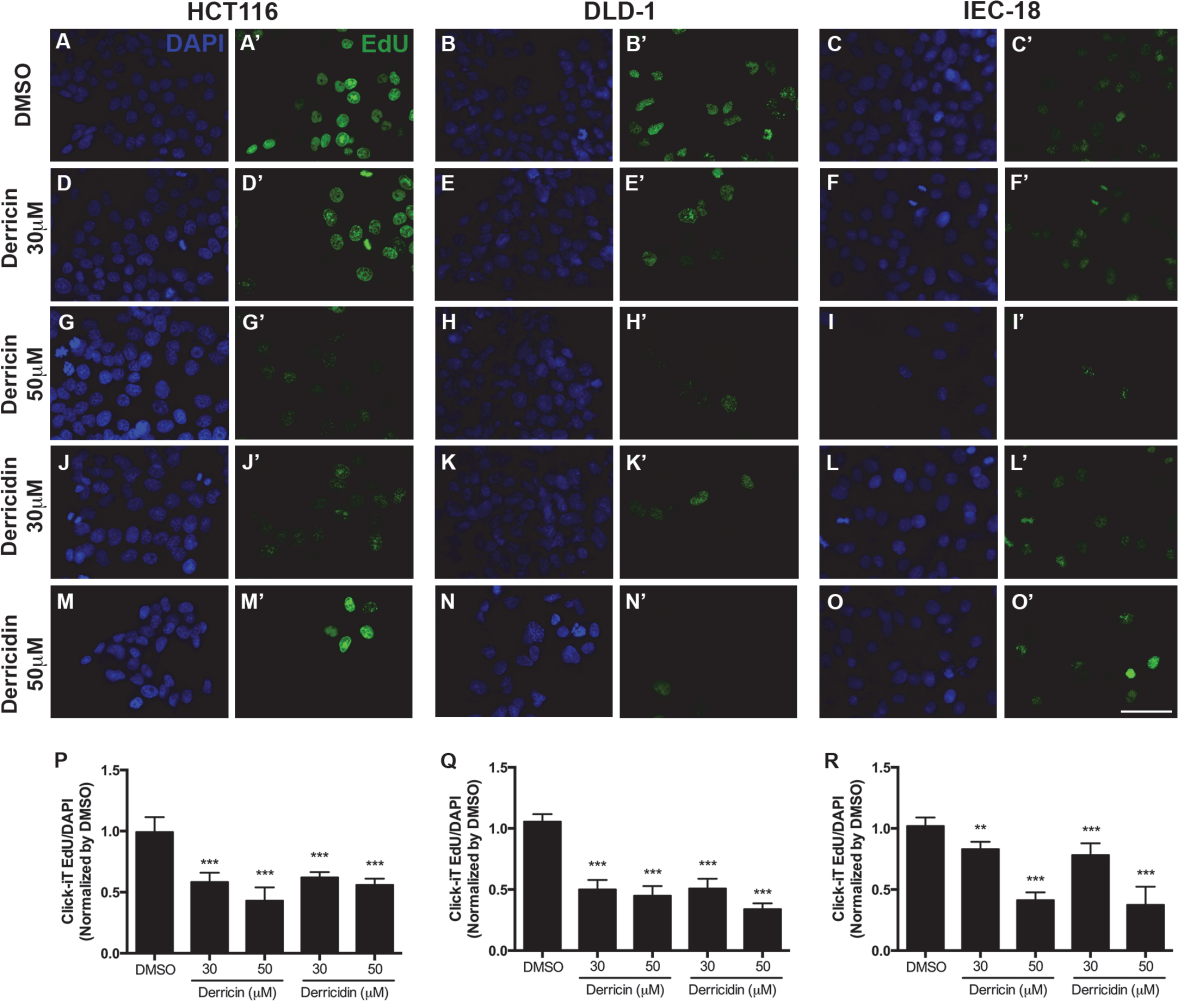
Derricin and derricidin inhibit cell proliferation of HCT116, DLD-1 and IEC-18 cells. Edu incorporation in colon cells after treatment with 30 and 50μM of derricin and derricidin for 24h. (A,A’,B,B’,C,C’) In control conditions, it is observed a considerable number of Edu-positive cells. (D,D’,E,E’,F,F’,G,G’,H,H’,I,I’) After treatment with derricin, it is observed a lower number of Edu-positive cells. (J,J’,K,K’,L,L’,M,M’,N,N’,O,O’) Derricidin treatment also reduces Edu-positive cells. (P,Q,R) Quantification of Edu-positive cells in HCT116, DLD-1 and IEC-18. Scale bar 50 μm, **p<0.01, ***p<0.001.

Our first analyses revealed that derricin and derricidin were able to decrease cell viability, suggesting that both flavonoids affect cancer cell growth. To test whether derricin and derricidin could inhibit proliferation of CRCs cell lines, we performed Edu-incorporation assay. We noted that both chalcones were able to reduce the number of proliferating cells, at 30 and 50 μM, in all cell lines (Fig. 3 A-O’). After quantification, it was possible to observe that 30 μM of both chalcones inhibit approximately 40% of cell proliferation in HCT116 and about 50% in DLD-1 (Fig. 3 P,Q). Interestingly, 30 μM of both flavonoids were able to inhibit only about 20% of cell proliferation in IEC-18, which indicate a difference in regulation of cell division of tumoral and non-tumoral cell lines (Fig. 3R).

**Fig 4:**
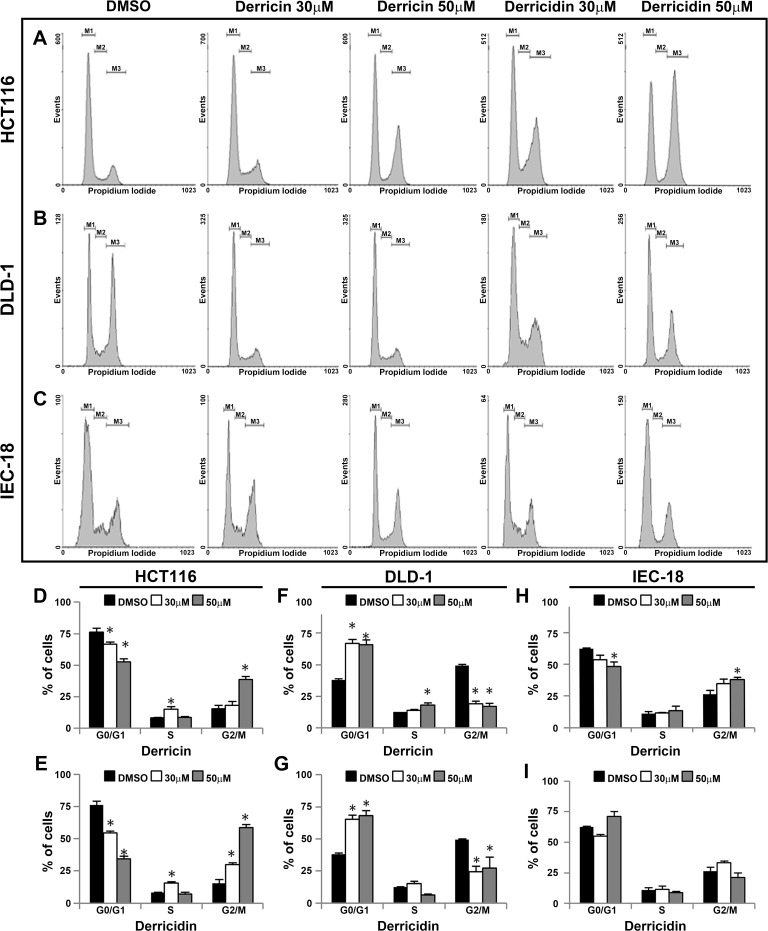
Derricin and derricidin induced cell cycle arrest in CRC cells. (A, D, E) In HCT116, untreated cells showed approximately 75% of cells in G1/G0 phase, while 30 and 50 μM of derricin reduced this proportion to 67.6% and 54%, respectively, resulting in cell cycle arrest in S and G2/M phases, respectively. Derricidin reduced this proportion to 53.8% and 35.5%, respectively and also induced cell cycle arrest in G2/M phase. (B, F,G) On the other hand, DLD-1 cells accumulated in G0/G1 phase of cell cycle after 30 μM and 50 μM of derricin or derricidin. (C, H, I) Derricin and derricidin treatment had milder effects in cell cycle progression of IEC-18 cells, except for a slightly arrest at G2/M with 50 μM of derricin. Bar graphs represent the percentage of cells according treatment and significant data were represented in the figure. Black bars: G0/G1 phase; White bars: S phase; and Gray bars: G2/M phases.

We next asked if these effects could be related to cell cycle arrest. To do this, we treated HCT116, DLD-1 and IEC-18 cells with each flavonoid for 96 h and quantified the percentage of cells in different cell cycle phases (Fig. 4). DMSO-treated HCT116 cells were mostly at G1/G0 cell cycle phase (Fig. 4A, in M1 region). Derricin treatment induced a significant disturbance in these diploid cells, which decreased cell count in the G1/G0 phase to 67.6% and 54% when treated with 30 and 50 μM of this flavonoid, respectively (Fig. 4A, in M1 region). Derricin affected the cell cycle in a dose-dependent manner, considering that 30 μM arrested the cell cycle in the S phase and 50 μM arrested the cell cycle in the G2/M phase (Fig. 4A,D). Derricidin was also evaluated for its ability to control the cell cycle. Treatment with 30 μM and 50 μM of derricidin reduced the percentage of diploid cells to 53.8% and 35.5%, respectively and induced a significant cell cycle arrest in the G2/M phase in both concentrations (Fig. 4E). The frequency of DLD-1 cells containing duplicated DNA content was approximately 50% in control cells and increased to 30.3% and 59.3% of cells after treatment with 30 μM and 50 μM of derricidin. On the other hand, living DLD-1 adenocarcinoma cells accumulated on G0/G1 phase of the cell cycle after 30 μM and 50 μM of derricin (Fig. 4B, in M1 region). 31,6% of cells were in G0/G1 phase in untreated or DMSO-treated conditions. This number achieves 66,9% and 65,7% of cells in 30μM and 50μM, respectively, of derricin. These results also indicate a non-dependent dose response of derricin treatment (Fig. 4F). Similar effects were observed when we treated these cell line with derricidin at both concentrations (Fig. 4B, in M1 region, 4G). Interestingly, IEC-18 presented a weak cell cycle phase modulation in both derricin and derricidin treatments, except for a milder effect at 50 μM of derricin (Fig. 4C,H,I). Taken together, these data show an anti-proliferative effect of derricin and derricidin, which acted by arresting the cell cycle in HCT116 and DLD-1 tumor cell lines.

### Derricin and derricidin as potent inhibitors of Wnt/β-catenin signaling

**Fig 5:**
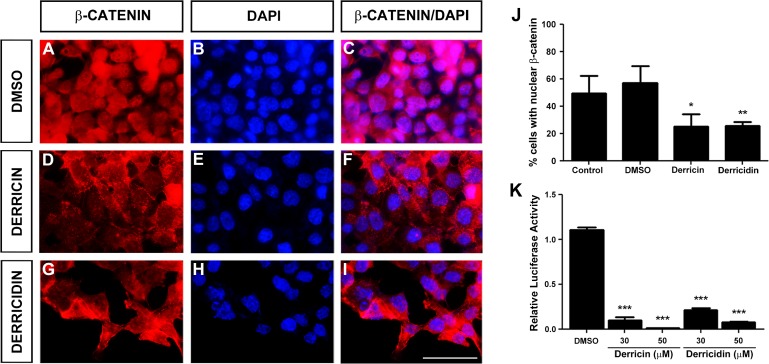
Derricin and derricidin affect β-catenin cell localization and Wnt reporter activity in HCT116 cells. (A-I) β-catenin immunostaining of HCT116 cells treated with 30 μM of each chalcone for 24 h. (A-C) In control conditions, majority of cells show β-catenin staining of nucleus. (D-F) After treatment with derricin, this staining is lost and most cells show β-catenin in cytoplasm and membrane. (G-I) Derricidin treatment also reduces nuclear staining of β-catenin. (J) Quantification of immunostaining showed that both chalcones reduce nuclear β-catenin. (K) Wnt/β–catenin specific reporter assay of TOPFlash/*Renilla* transfected HCT116 cells. Both chalcones reduce Wnt reporter activity after 30 and 50 μM treatment for 24 h. Scale bar: 50 μM, *p<0.05,**p<0.01, ***p<0.001.

Deregulation in canonical Wnt signaling is closely related to colon cancer cells [1,3]. For instance, HCT116 cells have a mutation in a CTNNB1 gene that encodes β-catenin protein, and thus have a high activity of the Wnt/β-catenin pathway [2]. We asked whether the cell viability and cell cycle-arrest effects of derricin and derricidin in HCT116 cells were due to modulation of Wnt/β-catenin signaling. We first analyzed the cellular distribution of β-catenin. In untreated or DMSO-treated cells, β-catenin protein was found in nuclei of nearly 50% of the cells (Fig. 5A, B,C,J). However, when we treated cells with derricin, only 25% of cells showed nuclear staining with β-catenin (Fig. 5D,E,F,J). In the case of derricidin, 26% of cells showed nuclear β-catenin, which was also a significant reduction in nuclear staining (Fig. 5G,H,I,J). Because these results support a possible Wnt/β-catenin inhibition, we addressed the Wnt pathway activation state more directly, by performing Wnt/β-catenin specific reporter assays on HCT116 transfected with TOPFlash plasmid. Consistently with previous data, derricin and derricidin specifically inhibited Wnt reporter activity (Fig. 5K). These results show that derricin and derricidin were able to inhibit the Wnt/β-catenin pathway in the HCT116 tumor cell line.

**Fig 6:**
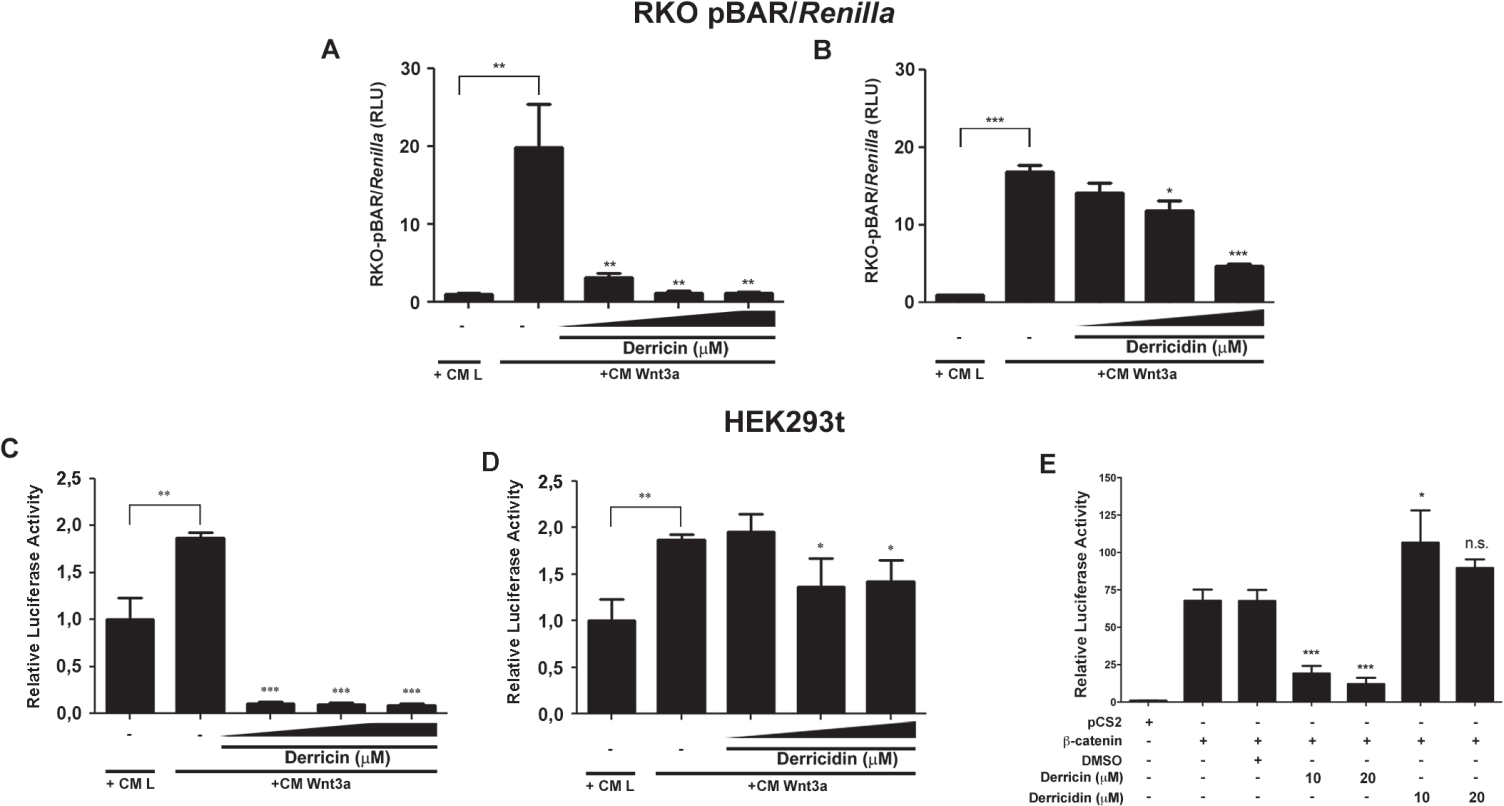
Derricin and derricidin inhibit Wnt-reporter activity in RKO-pBAR/*Renilla* and HEK293t. (A,C) Derricin inhibits Wnt-reporter activity starting at 10 μM in luciferase assays of RKO-pBAR/*Renilla* and of HEK293t. (B,D) Derricidin slightly inhibits Wnt-reporter activity at 20 μM in RKO-pBAR/*Renilla* and HEK293t. In RKO-pBAR/*Renilla*, higher inhibition is achieved at 50 μM of derricidin (B). (A,B) 10, 20, 50 μM of each chalcone. (C,D) 10, 20, 30 μM of each chalcone. (E) HEK293T activation of Wnt signaling through transfection of β-catenin is suppressed upon derricin treatment, but not derricidin. *p<0.05, **p<0.01, ***p<0.001.

Our experiments using HCT116 strongly suggest that derricin and derricidin are potent inhibitors of canonical Wnt signaling. To address this effect more specifically, we performed classical experiments to analyze Wnt pathway regulation, i.e., luciferase reporter-gene assays in RKO-pBAR/*Renilla* cells and HEK293t cells. These cells were treated with 10, 20 or 50 μM of derricin in the presence of Wnt3a conditioned medium. Derricin decreased the Wnt reporter activity in a concentration-dependent manner in both cell lines (Fig. 6A,C). Derricidin treatment was also able to decrease Wnt reporter activity, although the highest inhibition was only achieved when cells were treated with 50 μM (Fig. 6B,D). We observed a slight reduction in Wnt reporter activity after treatment with 20 μM derricidin. To know whether derricin and derricidin act upstream or downstream to the effector protein β-catenin, we activated the Wnt signaling by transfecting the HEK293t cells with β-catenin and evaluated the chalcones inhibition effects. Derricin was able to inhibit the β-catenin activation of Wnt pathway, whereas derricidin was not, suggesting that derricin acts downstream of β-catenin while derricidin acts upstream (Fig. 6E). These data show that derricin and derricidin behave as inhibitors of the Wnt/β-catenin signaling pathway, but in different concentrations and in different levels of this signaling.

### Derricin and derricidin inhibit double axis formation in *Xenopus* embryos and suppress Wnt/β-catenin luciferase reporter activity *in vivo*

**Fig 7:**
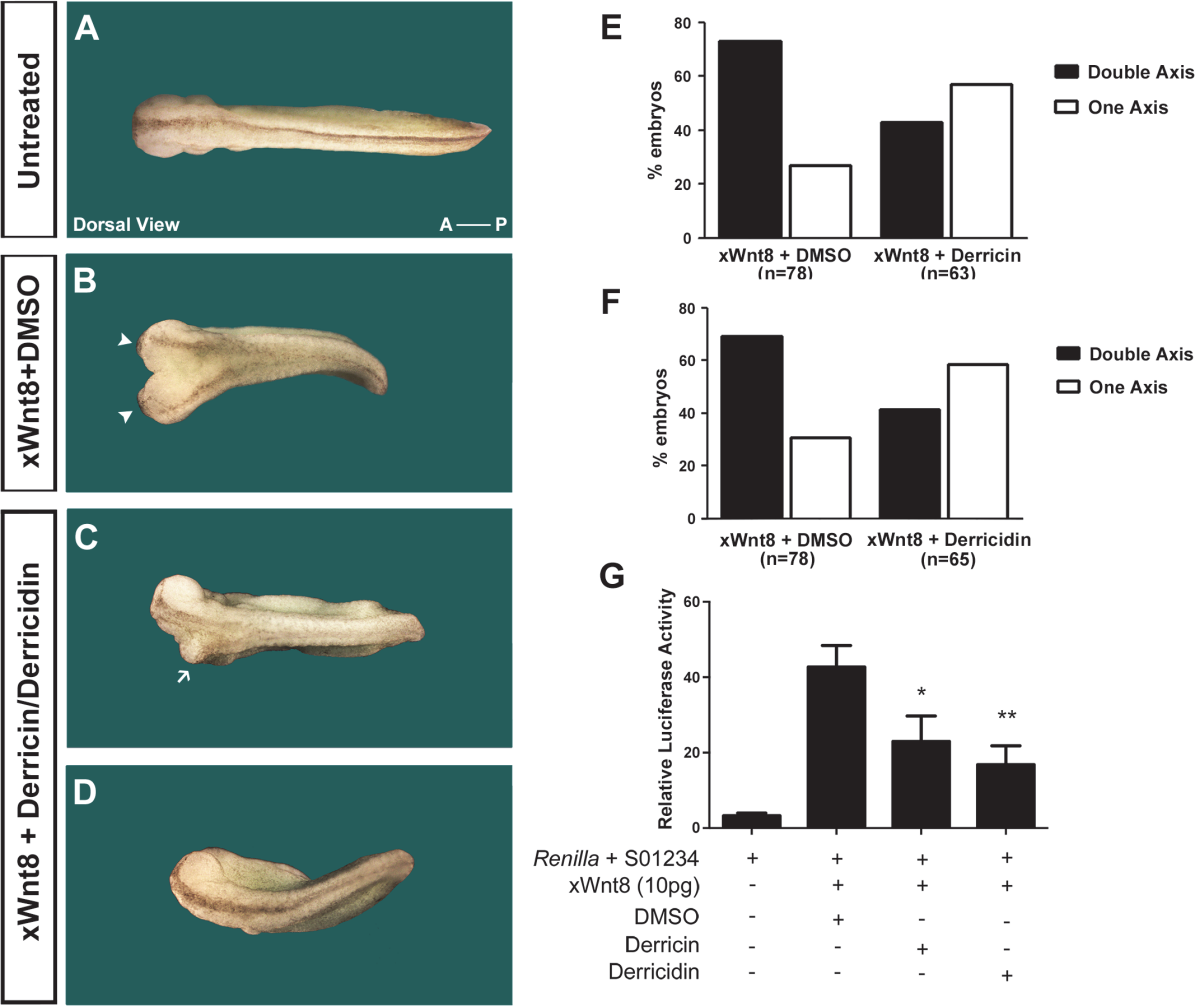
Derricin and derricidin inhibit double axis formation in Xenopus embryos and suppress Wnt/β-catenin luciferase reporter activity *in vivo*. Representative images of embryos co-injected with xWnt8 mRNA plus DMSO (B) or xWnt8 mRNA plus derricin or derricidin (C,D). Observe the incomplete double axis formation or secondary axis ablation in embryos injected with chalcones. (E-F) Graphs show percentages of embryo phenotypes. Observe that co-injection of chalcones with xWnt8 mRNA reduced the percentage of embryos with double axis, and increased the proportion of normal embryos, with one axis. (G) *Xenopus* embryos injected with S01234 reporter + *Renilla*, xwnt8 (10pg) with DMSO or the chalcones (0.4 pmol/embryo). The activation of the reporter was prevented by chalcone injection. Arrowheads: cement gland; arrow: incomplete double axis; A-P: anterior-posterior axis.

The Wnt-induced secondary axis formation in *Xenopus* is related to overactivation of Wnt/β-catenin signaling during development of *Xenopus* embryos, and thus, negative modulators of this pathway are able to reverse or reduce the double axis phenotype [26,27]. As our data strongly indicated that these flavonoids are inhibitors of canonical Wnt signaling, we decided to analyze the ability of derricin and derricidin to affect Wnt-induced double axis formation in *Xenopus* embryos. We co-injected embryos with *x*Wnt8 mRNA and derricin or derricidin in the ventral blastomeres of 4-cell-stage *Xenopus* embryos. We observed that approximately 73% of the embryos displayed a secondary axis after *x*Wnt8 injection (Fig. 7B,E,F). However, co-injection of *x*Wnt8 with derricin or derricidin reduced the induction of ectopic axis (Fig. 7C,D). Co-injection with derricin was able to reduce the double axis by 43%, while derricidin reduced it by 41% (Fig. 7E,F). In addition, we performed luciferase experiments by injecting the plasmid S01234-Luc at the ventral side of *Xenopus* embryos. This *siamois*-reporter is able to respond to the Wnt signaling activation induced by the co-injecting of xWnt8 mRNA. We injected concomitantly 0.4 pmol/embryo of derricin and derricidin to test whether they can inhibit the reporter activation. As a result, both chalcones reduced the reporter activity significantly (Fig. 7G). These data indicate that derricin and derricidin can also inhibit the Wnt/β-catenin pathway *in vivo* in the *Xenopus laevis* embryo model.

## Discussion

Flavonoid consumption has long been associated with prevention, control and treatment of various human cancers [28]. In regard to colorectal cancer, many flavonoids have been associated with the control and prevention of this disease [9,16–18]. Chalcones, for instance, may offer advantages as antitumor agents, compared with other flavonoids, because they have little interaction with DNA and thus a low risk of mutagenesis [29]. In this study, we showed that two flavonoids (Fig. 1), from the chalcone subclass, have potent anti-proliferative effects in colorectal tumor cell lines, HCT116 and DLD-1. Derricin and derricidin both strongly inhibited total cell count and viability of these cells *in vitro* (Fig. 2) and also reduced HCT116 and DLD-1 cell proliferation in similar levels after 24 h (Fig. 3). We also analyzed derricin and derricidin effects in IEC-18, a non-transformed epithelial cell line. Cell viability was only impaired at higher concentrations, differently of what occurs in HCT116 and DLD-1 cells. Moreover, 30μM of both chalcones had a slightly effect in IEC-18 cell proliferation, while a reduction of approximately 50% was observed in HCT116 and DLD-1 cells. These results suggest a possible effect more restricted to CRC cells.

Derricin and derricidin effects are related to modulation of the cell cycle progression, mainly by changing the percentage of cells in the G2/M and S phases of cell cycle in HCT116 and G0/G1 in DLD-1 (Fig. 4). Interestingly, derricin and derricidin cell cycle arrest occurs in different phases of cell cycle in each cell line. These effects can be related with a specific flavonoid effect in modulation of various cell cycle regulatory proteins, and differences in the background of mutated genes in each cell line can explain these distinct effects [30]. In addition, derricin and derricidin had no significant effects in IEC-18, a non-tumor cell line. These results can be correlated with other reports of important cytotoxic effects of these flavonoids [31,32]. Others chalcones possess apoptotic and antiproliferative effects [33,34,35].

Recent studies have improved understanding of the mechanisms of action of flavonoids. For instance, many flavonoids are modulators of different signaling pathways, such as Wnt/β-catenin, which is commonly associated with colorectal tumor progression [1,2,3,5,36]. In this study we observed that the antitumor effects of derricin and derricidin can be related to Wnt pathway modulation because they significantly reduced nuclear localization of β-catenin and reduced Wnt-reporter activation in HCT116 cells (Fig. 5). To better understand the effect on Wnt signaling in these chalcones, we performed gene-reporter assays in the RKO-pBAR/*Renilla* and HEK293T cell lines, which are classically used to investigate this pathway [21,26]. Derricin strongly inhibited Wnt signaling at a concentration of 10 μM, while derricidin reached similar inhibitory levels only at 50 μM in both cell lines (Fig. 6A,C). Also, derricin and derricidin inhibit Wnt signaling through different mechanisms, since derricin is capable of counteracting β-catenin Wnt signaling activation, while derricidin is not (Fig. 6E). This difference in the concentration-related effects of the two chalcones might be related to their chemical structures and/or to the way that they interact with Wnt components or cell compartments, which have profound effects on their potency and selectivity. Unfortunately, structure-activity relationships of chalcones and Wnt signaling modulation have not been addressed yet. However, a recent report showed that modification of the basic flavone core improves the selectivity and potency of flavones in Wnt/β-catenin modulation [37]. Similar studies have been performed with other natural substances and also synthetic compounds [38,39]. Moreover, we can observe once again differences of derricin and derricidin effects concerning cell line specificity. Regarding Wnt signaling, for instance, HCT116 and DLD-1 have different mutations in Wnt components, as β-catenin and APC, respectively, while in RKO and HEK293t these mutations are not present [2]. These mutations could be the basis of the peculiarity in biological effects of these chalcones and should be further investigated.

We also analyzed the *in vivo* effects of these chalcones on Wnt/β-catenin modulation in *Xenopus* embryos, which are widely used as an *in vivo* model to search for new Wnt regulatory molecules [26,27]. Derricin and derricidin effectively rescued the xWnt8-induced double axis, in comparison to the vehicle control (Fig. 7E,F). The proportion of rescued double-axis was similar to other Wnt pathway inhibitors previously described [27,40,41]. Also, the chalcones were capable of suppressing the xWnt8 induction of S01234 reporter in *Xenopus* embryo (Fig. 7G). In summary, these *in vivo* assays again demonstrated that these chalcones have potential effects on the modulation of the Wnt/β-catenin pathway.

Taken together, our results show for the first time that derricin and derricidin are potential modulators of the Wnt/β-catenin pathway. These results suggest questions about the mechanisms of action of these chalcones in Wnt/β-catenin pathway, specifically with which Wnt component they can interact. As derricin and derricidin are chalcones with very similar chemical structures, it is also interesting to investigate their differences in regard to structure-activity relationships. As chemical screenings of Wnt regulators based on structure-activity relationships are a recent issue in investigations of Wnt signaling, these important questions should be further addressed and will certainly open new routes toward understanding Wnt pathway interactions.

In conclusion, our results show that the Wnt pathway modulation exerted by derricin and derricidin make these chalcones promising candidates for the prevention or treatment of Wnt signaling-related pathologies, such as colorectal cancer.
